# An Optimization of Liquid–Liquid Extraction of Urinary Volatile and Semi-Volatile Compounds and Its Application for Gas Chromatography-Mass Spectrometry and Proton Nuclear Magnetic Resonance Spectroscopy

**DOI:** 10.3390/molecules25163651

**Published:** 2020-08-11

**Authors:** Natalia Drabińska, Piotr Młynarz, Ben de Lacy Costello, Peter Jones, Karolina Mielko, Justyna Mielnik, Raj Persad, Norman Mark Ratcliffe

**Affiliations:** 1 Institute of Animal Reproduction and Food Research of Polish Academy of Sciences, 10 Tuwima Str., 10-748 Olsztyn, Poland; 2 Institute of Biosensor Technology, University of the West of England, Coldharbour Lane, Frenchay, Bristol BS16 1QY, UK; Norman.Ratcliffe@uwe.ac.uk; 3 Department of Biochemistry, Molecular Biology and Biotechnology, Faculty of Chemistry, Wroclaw University of Science and Technology, 27 Wybrzeże Stanisława Wyspianskiego, 50-370 Wroclaw, Poland; piotr.mlynarz@pwr.edu.pl (P.M.); karolina.mielko@pwr.edu.pl (K.M.); justyna.mielnik@pwr.edu.pl (J.M.); 4 Indigo Science Ltd., Bristol BS7 9JS, UK; peter.jones@indigoscience.com; 5 Bristol Urological Institute, Southmead Hospital, Bristol BS10 5BN, UK; rajpersad@bristolurology.com

**Keywords:** liquid–liquid extraction, volatile compounds, urine, method optimization, GC-MS, ^1^H-NMR

## Abstract

Urinary volatile compounds (VCs) have been recently assessed for disease diagnoses. They belong to very diverse chemical classes, and they are characterized by different volatilities, polarities and concentrations, complicating their analysis via a single analytical procedure. There remains a need for better, lower-cost methods for VC biomarker discovery. Thus, there is a strong need for alternative methods, enabling the detection of a broader range of VCs. Therefore, the main aim of this study was to optimize a simple and reliable liquid–liquid extraction (LLE) procedure for the analysis of VCs in urine using gas chromatography-mass spectrometry (GC-MS), in order to obtain the maximum number of responses. Extraction parameters such as pH, type of solvent and ionic strength were optimized. Moreover, the same extracts were analyzed using Proton Nuclear Magnetic Resonance Spectroscopy (^1^H-NMR), to evaluate the applicability of a single urine extraction for multiplatform purposes. After the evaluation of experimental conditions, an LLE protocol using 2 mL of urine in the presence of 2 mL of 1 M sulfuric acid and sodium sulphate extracted with dichloromethane was found to be optimal. The optimized method was validated with the external standards and was found to be precise and linear, and allowed for detection of >400 peaks in a single run present in at least 50% of six samples—considerably more than the number of peaks detected by solid-phase microextracton fiber pre-concentration-GC-MS (328 ± 6 vs. 234 ± 4). ^1^H-NMR spectroscopy of the polar and non-polar extracts extended the range to >40 more (mainly low volatility compounds) metabolites (non-destructively), the majority of which were different from GC-MS. The more peaks detectable, the greater the opportunity of assessing a fingerprint of several compounds to aid biomarker discovery. In summary, we have successfully demonstrated the potential of LLE as a cheap and simple alternative for the analysis of VCs in urine, and for the first time the applicability of a single urine solvent extraction procedure for detecting a wide range of analytes using both GC-MS and ^1^H-NMR analysis to enhance putative biomarker detection. The proposed method will simplify the transport between laboratories and storage of samples, as compared to intact urine samples.

## 1. Introduction

Modern metabolomics is now a high-throughput approach for the monitoring of metabolites in biological tissue or fluid in a defined time point. The profile of metabolites may vary during pathological states, hormonal changes, exposure to environmental pollutants, diet, etc., and the changes can be determined in different biological specimens, such as urine, saliva, blood, skin, feces, breath and sweat [1,2]. Urine, because of the non-invasive methods of collection and the richness of metabolites, is a commonly used fluid in metabolite profiling [3,4], potentially giving a large amount of information about the metabolic state of the body. Urine is frequently analyzed using Liquid Chromatography-Mass Spectrometry (LC-MS), Gas Chromatography-Mass Spectrometry (GC-MS) and Nuclear Magnetic Resonance Spectroscopy (NMR) [5,6,7]. A specific class of metabolomics focused on the profile of volatile compounds (VCs) is termed volatolomics, the applications of which for diagnostic purposes is growing [8,9,10,11,12,13,14,15]. VCs are secreted by cells of the human body, as a result of their metabolism. The changes in the profile of VCs in biological fluids, dependent on the metabolic changes, may reflect the presence of disease. Many studies have suggested that the profile of VCs in urine change in cancer [8,9,10], nephrological conditions [15], oxidative stress [11], gastrointestinal diseases [12,13,14] and other disease states.

VCs in urine belong to very diverse chemical classes, such as aldehydes, ketones, organic short chain acids, alcohols, sulfur compounds, etc. They are characterized by different volatilities, polarities and concentrations. These facts complicate the optimization of conditions for VC profiling in a single analytical procedure. To date, many analytical approaches have been used for the analysis of urinary VCs, such as sensor systems [16], Field Asymmetric Ion Mobility Spectrometry (FAIMS) [12], as well as hyphenated techniques based on mass spectrometry, such as High-Pressure Photon Ionization Time-of-Flight-Mass Spectrometry (HPPI-ToF-MS) [17], Proton Transfer Reaction-Mass Spectrometry (PTR-MS) [18], Selected Ion Flow Tube Mass Spectrometry (SIFT-MS) [19], and finally the most ubiquitous technique, GC-MS [10]. The latter is still considered the gold standard in VC analysis.

Direct headspace methods of urine sampling suffer from low sensitivity, which hinders their diagnostic potential for disease diagnoses. An analysis of VCs with GC-MS typically requires a sample preparation step, particularly pre-concentration of analytes. These sample preparation methods for VC analysis in urine are comprised mostly of adsorption techniques, such as solid-phase microextraction (SPME) [8,10,20,21,22] and thermal desorption with sorbent tubes [23]. These are selective techniques, which are also adversely affected by high water concentrations, limiting the number of compounds detected and reducing their usefulness for non-targeted profiling. Therefore, there is a strong need for alternative methods that enable the detection of a broader range of compounds.

VCs can be extracted from the matrix using more conventional approaches. For example, liquid–liquid extraction (LLE) is a traditional and favored extraction technique in analytical chemistry, because of its simplicity and lack of complicated equipment. LLE is used in a wide range of applications and for the extraction of varied classes of compounds in food chemistry [24,25], environmental analysis [26], drug analysis [27], etc. Even though LLE is so commonly used in analytical chemistry and industry, its application for the extraction of VCs from biological fluids is not as frequent as would have been expected [28]. The extraction of individual VCs has been proposed by Seyler et al. [29], who optimized the method for quantification of six nirosamines in urine. LLE potentially permits relatively high concentrations of a diverse range of VCs to be attained, providing a low-cost simple alternative to more expensive complex extraction technologies, while extracting VC and semi-VCs with a range of polarities.

From the metabolomic viewpoint, it is also important to obtain extracts suitable for analysis utilizing a more diverse range of approaches. The application of different analytical techniques combining GC-MS and ^1^H-NMR gives the possibility of determining a broader set of metabolites, with varying volatility, from VCs to semi-VCs, including non-polar compounds. The main aim of this study was to optimize a simple and reliable LLE procedure for VCs and semi-VCs analysis in urine using GC-MS. To do so, extraction parameters, such as pH, type of solvent and ionic strength, were optimized by considering the maximum number of deconvoluted peaks detected. Next, the optimized method was validated in term of precision, linearity and sensitivity. Moreover, the same extracts were analysed using ^1^H-NMR, to evaluate for the first time the applicability of a single urine extract for multiplatform purposes, which should increase the prospects for linking urine metabolites to a particular disease. The particular purpose of the study is to show the enhanced number of (uncharacterized) compounds found using LLE, relative to other methods.

## 2. Results and Discussion

### 2.1. Optimization of the Extraction Parameters

In the present study, the type of solvent, acid molarity and ionic strength were selected and evaluated to achieve the optimal condition of LLE, based on the maximum number of GC-MS peaks detected (Table 1). The solvents, acidic pH and amount of salt were preselected based on data from the literature [28,29,30].

Dichloromethane (DCM) was found to be the most efficient solvent for VC extraction from urine samples, followed by chloroform (Table 1 and Supplementary Figure S1). DCM is immiscible with water and can dissolve a wide range of organic compounds, hence its extensive use for LLE [31,32]. Diethyl ether is a commonly used solvent for extracting organic compounds from aqueous solutions, however in this study on urine, it was found to be the least effective solvent for LLE, resulting in the detection of only circa 20 peaks. In contrast, Zlatkis et al. [28] noted the presence of 300 compounds in ether extracts (comparison to the efficiency of other solvents was not analyzed), out of which 40 have been identified. However, in their study, a very large amount of urine, 450 mL, was used for extraction with 80 mL of diethyl ether, as compared to 2 mL of urine used in our study. Use of 450 mL urine creates storage issues, and many patients could not produce such an amount. The use of 80 mL of ether for extraction requires more processing time, i.e., more drying agent, evaporation times, and it is undesirable from the health and safety perspective and impractical for certain processing steps such as centrifugation. 2 mL is a realistic volume of urine from a patient sample, which can be collected clinically, therefore the present study was optimized using that volume. In comparison to our methodology, the literature describes VC extraction using absorbents, and smaller numbers (75 and 147 peaks identified, respectively) of VCs were reported, even with larger amounts of urine (using single quadrupole GC-MS) [22,30].

In the present study, the highest molarity of acid (1 M) with pH value near 0 was found to be significantly more efficient for LLE (Table 1). There was no significant difference between 0.1 M (pH value: ~1.5) and 0.01 M (pH value: ~2.5) acid addition. Acidic conditions were previously reported to be more suitable for VC analysis as compared to basic and neutral pH when using SPME [4,14,30]. This is related to the chemical properties of the compounds present in urine. Acidic pH increases the number of compounds in the non-conjugated form [4]. In our study, many of the VCs detected in urine samples contained the carboxylic acid group, therefore the acidic pH facilitates their extraction [21].

Another parameter important to achieving a good extraction is the presence of salt. The results of the experiments carried out with and without salt present are presented in Table 1. The presence of salt changes the nature of the molecular interactions between compounds, causing more ionic activity, and consequently affecting the activity coefficient of metabolites. It was found to have great importance in the SPME method’s development, where the presence of salt facilitates the transfer of VCs from the matrix to the headspace [33]. For LLE, salt addition may alter the solubility of certain compounds in the matrix, making them more likely to transfer to the solvent. In the present study, as in LLE, the transfer of VCs to the headspace is not needed, and we did not observe a statistically significant difference in the number of peaks between the presence and absence of salt. However, taking into consideration the slightly increased number of peaks detected (even though not significant) and their size (a summarized peak area of 5.49 × 10^5^ vs. 1.09 × 10^6^ for no salt and salt addition, respectively), we decided to carry out the experiments with the presence of sodium sulphate. It is worth underlining that the urine contains salt, the concentration of which may vary from sample to sample, affecting the results. Therefore, it is important to standardize the urine somehow, e.g., by the analysis of the osmolality of urine samples before metabolomics analyses. In our method, the saturation of urine with salt was achieved, minimizing the differences between samples.

**Figure 1:**
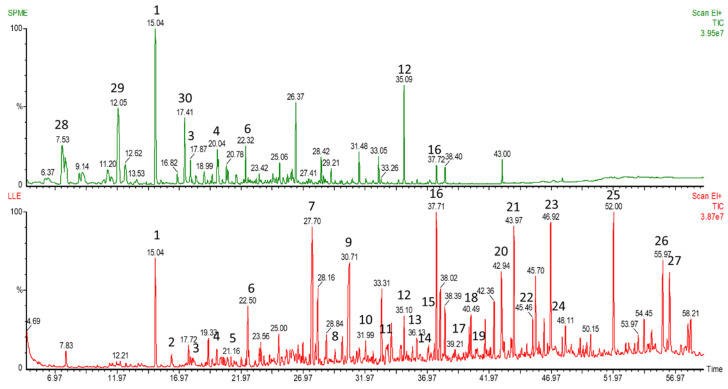
The comparison of total ion mass chromatograms of volatile compounds (VCs) extracted from urine sample using solid-phase microextraction (green chromatogram) and the optimized method (red chromatogram). The tentatively identified compounds: (**1**) Allyl isothiocyanate; (**2**) Tetradecane; (**3**) Acetic acid; (**4**) 2-Butyl-1-octanol; (**5**) Diethyl sulfoxide; (**6**) hexadecane; (**7**) tetrahydro-6-methyl-2*H*-Pyran-2-one; (**8**) 2-Methoxy-phenol; (**9**) Dimethyl sulfone; (**10**) Heptanoic acid; (**11**) 2-Methyl-octanoic acid; (**12**) *p*-Cresol; (**13**) Erucin; (**14**) Nonanoic acid; (**15**) Octenoic acid; (**16**) 2-Methoxy-4-vinylphenol; (**17**) *n*-Decanoic acid; (**18**) Divinyl sulphide; (**19**) 1-Hexadecanol; (**20**) Benzoic acid; (**21**) 7-Methylindole (**22**) Benzeneacetic acid; (**23**) Apocynin; (**24**) Benzamide; (**25**) *n*-Hexadecanoic acid; (**26**) Octadecanoic acid; (**27**) Caffeine; (**28**) 4-heptanone; (**29**) *p*-Cymene; (**30**) 4-Ethenyl-1,2-dimethylbenzene.

After the evaluation of experimental conditions, the best conditions for the LLE extraction of VCs from urine samples were as follows: 2 mL of 1 M sulfuric acid and 2 mL of urine was added to a 10 mL glass vial containing 0.2 g of sodium sulphate. The vial was vortexed till complete salt dissolution. Then, 4 mL of DCM was added and mixed again for 1 min. After that, the vial was centrifuged for 1 min at 3500 rpm for emulsion separation, and the DCM layer was collected and dried with anhydrous sodium sulphate. 3 mL of the dried DCM extract was quantitatively transferred to a new 5 mL glass vial for evaporation. The last approximately 100 μL of extract was then transferred to a GC vial, and the previously used 5 mL vial was rinsed three times with approximately 100 μL of fresh DCM. After the evaporation to dryness in the heating dry block at 40 °C, the residue was reconstituted in 10 μL of DCM, and 2 μL of the extract was injected into the GC injector port. An example of the chromatogram obtained using the optimized method is presented in Figure 1 (red chromatogram).

### 2.2. Analytical Performance

The demonstration that the method is of high quality is a crucial step in method development [34]. The validation of the method for non-targeted metabolomics comprises in most cases solely the analysis of precision [2]. Therefore, to check the reliability of the methodology, an external standard method was applied. Seven commercial standards, representing acids and aldehydes with different chain lengths, were selected, and the standard mixture was used for precision, sensitivity and linearity evaluation. The results are presented in Table 2.

The intraday and interday precision ranged from 6.1% to 14.9%, and from 9.1% to 26.4%, respectively. Naz et al. [34] recommended that the Relative Standard Deviation (RSD) values should not exceed 30% in metabolomics studies, therefore the results obtained in our study can be considered satisfactory, especially for the manual injection applied in our study. The correlation coefficients (R^2^) of the calibration curves for all selected standards ranged between 0.98 and 0.99, indicating that the method has a highly linear response for the concentration ranges presented in Table 2. The limit of detection (LOD) and limit of quantification (LOQ) values were found to be less than 10 and less than 33.3 μmol/L, respectively, for all the analyzed standards. However, the sensitivity of the method was not the priority of this study.

### 2.3. Method Application—GC-MS

The applicability of the optimized method was evaluated based on the analysis of six urine samples collected from healthy individuals. The comparison of the samples was not the aim of this study, therefore the urine samples were not normalized. However, for the application of the method in metabolomics studies in the future, the normalization will be necessary [35]. The analysis of urine with more analytical techniques can increase the number of metabolites of different physicochemical properties, and consequently may provide more information about the metabolic state of the body [36]. Therefore, in the present study, we decided to conduct the analysis of the samples using both GC-MS and ^1^H-NMR spectroscopy, with the aim of detecting greater numbers of peaks corresponding to individual compounds from a single extract.

The GC-MS analysis resulted in a total number of 400 individual deconvoluted peaks representing different compounds, detected in chromatographs, present in at least 50% of samples. The number of VCs detected in each sample is presented in Table 3. A comparison between the optimized method and SPME method is presented in Figure 1. The extraction of VCs from another individual run using both LLE and SPME of a headspace above the urine from the same individual showed a greater number of deconvoluted peaks detected using our optimized method (328 ± 5.66 vs. 234 ± 4.24, for LLE and SPME, respectively). As can be seen in Figure 1, the optimized LLE method results in more peaks at longer retention times, corresponding to heavier molecules, when compared to the SPME method, which is more efficient for small mass VCs. A comparison of the number of peaks detected by our method and those in the literature using SPME fiber pre-concentration technology with urine shows there is a significant improvement using LLE. The extraction using SPME with Divinylbenzene/Carboxen/Polydimethylsiloxane (DVB/CAR/PDMS) fiber and acidic conditions resulted in the detection of 75 VCs in urine in a single run [30]. In another study, a total number of 147 VCs was detected in urine using SPME with CAR/PDMS fiber; however, this number represents a sum of VCs obtained in acidic, basified and neutral pH samples [22]. On the other hand, in the study of Rocha et al. [37], GCxGC-TOFMS analysis allowed for the detection of approximately 700 compounds, of which 294 were tentatively identified, however it resulted from using a very high-cost and complex chromatographic system. Previous attempts at the application of LLE with diethyl ether using 450 mL of urine for VCs analysis resulted in the detection of 300 VCs, 40 of which were identified [28]. The results obtained proved that the optimized method is applicable for the GC-MS profiling of VCs in the urine.

### 2.4. Method Application—^1^H-NMR

**Figure 2:**
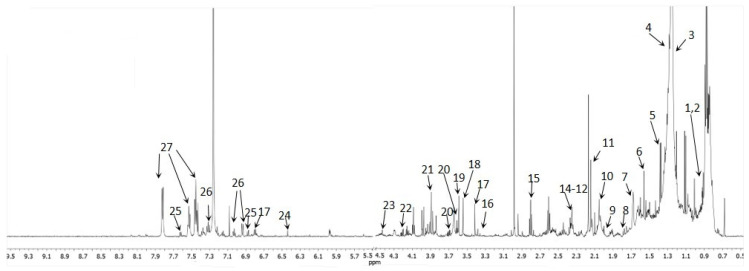
^1^H-NMR 600 MHz Carr–Purcell–Meiboom–Gill (CPMG) spectra of urine obtained from non-polar phase sample (CDCl_3_, T = 300 K); **1**—Cholesterol ester, **2**—Terminal -CH_3_, **3**—Acyl chain C4-C7, **4**—-(CH_2_)_n_-, **5**—2-hydroxyisobutyric acid, **6**—Saturated C3 acyl chain, **7**—-CO-CH_2_-CH_2_, **8**—Glycocholic acid, **9**—Acetamide, **10**—Allylic methylene -C=C-CH_2_, **11**—O-Acetylcarnitine, **12**—3-hydrixyisovaleric acid, **13**—Pyruvic acid, **14**—Acyl chain C2, **15**—Succinylacetone, **16**—Theophylline, **17**—3,4 Dihydroxybenzeneacetate, **18**—Phenylacetate, **19**—Glycine, **20**—Glycerol, **21**—Glycolic acid, **22**—Sn1+Sn3 -CH_2_-O-CO-R, **23**—1,3 dihydroxyacetone, **24**—Fumaric acid, **25**—Xanthurenic acid, **26**—Phenol derivative, **27**—Benzoic acid.

^1^H-NMR analysis was performed in both extract parts, because the information obtained is not only complementary but also supportive to some of the metabolites from GC-MS. The ^1^H-NMR spectra of the polar phase revealed approximately 60 proton signals originating from organic compounds within the urine. The signals are mostly located in the three areas of chemical shifts (δ): (a) 0.77–1.94 ppm characteristic for CH_3_ and CH_2_ groups; (b) 1.95–4.33 ppm for the aliphatic proton signal of different CH and CH_2_ groups, and (c) 6.8–8.07 ppm, distinctly visible peaks which in principle can exhibit protons originating from aromatic compounds. However, as is seen in the spectra (Figure 2), the signals from higher mass molecules are not filtered off completely by the CPMG pulse sequence. The signals in the polar fractions were assigned to the Chenomx references, where over 100 signals were detected, and among them 42 metabolites were found to be present in the urine aqueous phase (Supplementary Data Table S1). Interestingly, there were significant differences between the compounds tentatively identified by both GCMS and ^1^H-NMR, which proves that simultaneous analysis of the same extracts with these two methods is complementary.

The DCM (LLE) can extract metabolites with a range of polarities (particularly relatively non polar compounds), however it would not be expected to be that efficient in extracting polar compounds, such as ionic compounds [38]. The literature data related to non-polar compound analysis from biological fluids and tissues is very limited, usually including on the ^1^H NMR spectra a general description of the groups of compounds [39]. In another report, the urine extracts were dissolved in different deuterated solvents (MeOD, DMSO, DMF, MeCN, Acetone, CDCl_3_ and DCM), with subsequent monitoring of the levels of five metabolites: hippurate, creatinine, lactate, histidine and alanine [38]. Metabolites showed signal variation in the ppm scale depending on the solvent used. However, among the selected metabolites, only hippurate was found to be resolvable in CDCl_3_.

The study did not avoid some limitations. First, the method was optimized only for GC-MS, not both GC-MS and ^1^H-NMR. However, the main aim of the study was the optimization of LLE extraction for VC analysis, and the ^1^H-NMR part was the additional attempt, performed to check if the same extract can be used on multiple platforms. A second limitation is the small number of samples used for applicability testing. It is related to the character of the study, which is method development. The real urine sample analyses were included only to prove that the method is suitable for real sample analysis. Finally, the study does not contain the identification of all the compounds (however the main chemical groups were tentatively identified and mentioned). However, the authors decided not to undertake this exhaustive analysis because the main goal of this study was to detect the highest number of peaks corresponding to individual compounds that could be potential biomarkers. The identification would be scientifically interesting when defined peaks are identified as biomarkers linked to specific diseases. This will be the target for future studies using the developed LLE method.

## 3. Materials and Methods

### 3.1. Chemicals and Preparation of Calibration Solutions

The following analytical standard grade commercial chemicals were used: heptanal, octanal, hexanoic acid from Aldrich Chemicals (Milwaukee, WI, USA), and nonanal, decanal, heptanoic acid and octanoic acid from Acros Organics (Geel, Belgium). Sodium sulphate, DCM, chloroform and diethyl ether were purchased from Fisher Scientific (Hampton, NH, USA) and sulfuric acid was purchased from Aldrich Chemicals (Milwaukee, WI, USA),

The stock solution incorporating standards was prepared by dissolving 1 μL of each standard in MilliQ water (Millipore, Bedford, MA, USA) in a 100 mL measuring flask. The working solutions were prepared by diluting the standard stock solution, in the range 3 to 80 μmol/L.

### 3.2. Urine Samples

For method development, approx. 50 mL of morning urine sample was collected from one apparently healthy female volunteer, who had an ad hoc omnivore diet. The sample was immediately divided into 2 mL aliquots and stored in the fridge at 4 °C until the analysis which was conducted the same day.

For comparison of the LLE to the SPME method, a urine sample from one apparently healthy male volunteer was collected. The sample was treated as described above.

For method application, urine samples from six healthy individuals have been used. Samples were obtained from Liverpool Bio-Innovation Hub (LBIH) Biobank. The LBIH Biobank has Research Tissue Bank status and is licensed by the Human Tissue Authority (HTA). The collection and storage of biosamples has been ethically approved by the North West 5 Research Ethics Committee. Samples were stored in 2 mL aliquots at −80 °C after collection, and defrosted in the fridge at 4 °C before analysis. 

All procedures involving human participants were performed in accordance with the 1964 Helsinki Declaration and its later amendments or comparable ethical standards.

### 3.3. Extraction Optimization

The highest number of individually defined and chromatographically resolved peaks corresponding to VCs across the entire GC chromatogram was used as a measure of the best extraction performance conditions during method development. The deconvoluted peaks, subtracted from the blank samples, were counted.

**Figure 3:**
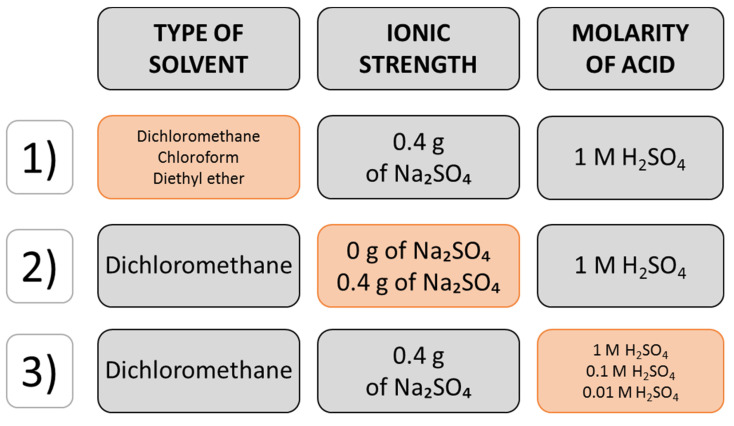
Flow diagram of the one-factor-at-a-time design of extraction optimization. The orange chart refers to conditions which were compared at a time.

Type of solvent (DCM, chloroform, diethyl ether), ionic strength (0 or 0.4 g of anhydrous sodium sulphate) and the molarity of sulfuric acid (0.01, 0.1 and 1 M) were investigated for their effect on extraction efficiency as presented in Figure 3. The extractions were performed by adding 2 mL of acid solution to 2 mL of urine and 0.2 g of anhydrous sodium sulphate, followed by vortexing until the salt dissolved. Next, 4 mL of organic solvent was added and vortexed for 1 min. The layers were separated by centrifugation for 1 min at 3500 rpm using a Beckman Coulter Aliegra X-22R Centrifuge (Brea, CA, USA). The solvent layer was dried with anhydrous sodium sulphate, collected and evaporated to dryness in a dry heating block at 40 °C for approximately 30 min. The dry residue was stored in a freezer at −20 °C until analysis and then dissolved in 10 μL of organic solvent used for the extraction. A 2 μL aliquot of the extract was immediately injected manually into the inlet of a gas chromatograph.

### 3.4. Analytical Performance

The optimized LLE procedure was validated in terms of precision, linearity and sensitivity based on the peak areas of heptanal, octanal, nonanal, decanal, hexanoic acid, heptanoic acid and octanoic acid used as external standards. The standard solutions were extracted using the optimized method. Precision was calculated and expressed as the RSD of six replicates of an aqueous stock standard solution with a concentration of 0.01 μL/mL (interday precision), followed by repeating the interday precision the next day (intraday precision). The linearity was determined by evaluation of the regression curves of the standard peak areas versus the concentration and expressed as the squared determination coefficient R^2^. The linear ranges were obtained by creating the calibration curves using six sequential dilutions of the working standard solutions. Sensitivity expressed as LOD and LOQ was calculated based on the signal-to-noise ratio (S/N). LOD was defined as the lowest concentration with a S/N ratio of 3, whereas the LOQ used a S/N ratio of 10.

### 3.5. SPME

The SPME method was performed according to the method described by Silva et al. [40] with slight modifications. Briefly, 2 mL of urine, 0.2 g of sodium sulphate and 2 mL of 1 M sulfuric acid were added to 20 mL headspace vial. The extraction was conducted manually by inserting the CAR/PDMS fiber into the sample vial and exposing it for 60 min at 50 °C. After the extractions, the fiber was introduced into the GC inlet and the VOCs were thermally desorbed for 10 min at 245 °C in a splitless mode.

### 3.6. Gas Chromatography-Mass Spectrometry

The analysis of urinary VCs was performed using a Hewlett Packard HP5890 series II GC coupled to an HP5971, single quadrupole Mass Selective Detector (MSD) with an HP Chemstation (Hewlett Packard, Bracknell, UK). Chromatographic separation was performed on a Stablewax DA capillary column, 30 m × 0.25 mm × 0.25 μm (Restek, Benner Circle, Bellefonte, PA, USA). The carrier gas was 99.9995% pure helium (AirProducts, Crewe, UK) with a constant flow rate of 1.2 mL/min. The GC was operated under the following conditions: temperature program, 40 °C with 2 min of hold time, ramping at 4 °C/min to the final temperature of 245 °C and then held at 245 °C for 6 min, giving a total run time of 59.25 min. The injection port was operated in splitless mode (purge off 0.7 min) at 245 °C. After a solvent delay of 4.6 min, mass spectra were acquired in full scan mode with a scan range of *m*/*z* 35–450 used for data acquisition. The operating conditions for the MS system were as follows: electron ionization mode at an energy of 70 eV; transfer line and ion source temperatures were 280 °C and 180 °C, respectively. Total ion chromatograms (TIC) were analyzed and peaks were integrated automatically using the Turbomass software (PerkinElmer, Inc., Waltham, MA, USA) with an initial detection threshold of 10.0. The analyst reviewed the automated integration and made adjustments and manually integrated the peaks, if it was necessary, keeping the proper judgement. Moreover, the chromatograms were analyzed using the free Automated Mass Spectral Deconvolution and Identification System (AMDIS) software by the National Institute of Standards and Technology (NIST, Gaithersburg, MD, USA). The deconvoluted peaks were tentatively identified, where possible, by comparison of the mass spectra with the NIST/EPA/NIH Mass Spectral Library (version 2.2, 2014, Gaithersburg, MD, USA). For identification, only the components with a match factor >80% were listed.

### 3.7. ^1^H-NMR

The urine extracts (organic and polar phases) were dissolved in 0.55 mL deuterochloroform (with an internal standard, TMS) and ^1^H-NMR spectra of the urine samples were recorded using an Avance II spectrometer (Brucker, Billerica, MA, USA) that was operating at a proton frequency of 600.58 MHz. The ^1^H-NMR spectra were collected using standard one-dimensional Carr–Purcell–Meiboom–Gill (CPMG) pulse sequence with water presaturation, at 300 K temperature. For each sample, 128–512 consecutive scans (NS) with a 400 μs spin-echo delay were collected; there were 80 loops for the T2 filter, with a 3.5 s relaxation delay and a 2.73 s acquisition time, a time-domain of 64k, and a spectral width of 20.02 ppm. The spectra were processed with a line broadening of 0.3 Hz and were manually phased and baseline corrected using Topspin 1.3 software (Brucker, Billerica, MA, USA). The water spectrum region was removed from the analysis.

### 3.8. Statistical Analyses

All the analyses were performed in triplicate. Data are presented as a mean ± standard deviation (SD). The data were compared with the one-way analysis of variance (ANOVA) test or Student *t*-test, as appropriate using the Statistica 10.0 software (StatSoft, Tulsa, OK, USA). Fisher’s Least Significant Difference (LSD) test was applied to assess significant differences (*p* < 0.05) between variables.

## 4. Conclusions

In conclusion, a simple, LLE-based sample preparation protocol for the metabolic profiling of urine samples was optimized and validated. The method was reliable and does not require specific instrumentation for sample preparation. We successfully demonstrated for the first time the applicability of single urine solvent extracts for both GC-MS as well as ^1^H-NMR analysis, for volatile and semi-volatile compound analysis. The possibility of obtaining a wider range of the metabolites can give more information about the health of the individuals, and may facilitate the identification of biomarkers linked to different diseases. The possibility of using both GC-MS and ^1^H-NMR platforms allowed the detection of metabolites with different volatilities from a single sample. This may be useful when carrying out future studies aimed at identifying metabolites linked to disease. Often, previous studies have had limited scope and concentrate on a limited range of methods. Using these methods, the detection capability could be increased many-fold by simply increasing the extraction volumes used; for instance, 40 mL DCM and 20 mL urine. The benefit of the proposed method is the possibility of storage of extracts in smaller vials, as compared to intact urine samples, and the possibility of transport to different labs for analysis in a dry way, without the special freezing conditions.

Future work will involve the application of the optimized method to look for diagnostic markers of urological cancers. In summary, LLE has been shown to be superior to the SPME fiber pre-concentration methodology for urine analyses, permitting semi-volatiles to be detected and providing a promising alternative methodology to the use of very costly absorptive technologies.

## Figures and Tables

**Table 1:** Comparison of the factors affecting the extraction efficiency of urine VCs using GC-MS analyses. Values in bold were selected as the most efficient and were used for the optimization of the next parameters.

Factor Analyzed	Number of Peaks Detected	Constant Conditions
Type of solvent (4 mL added)		
DCM *	205.0 ± 46.1 ^a,^**	1 M acid, salt addition
Chloroform	121.0 ± 47.6 ^b^	
Diethyl ether	20.7 ± 4.0 ^c^	
Acid molarity (2 mL added)		
0.01 M	137.0 ± 2.7 ^b^	solvent: DCM, salt addition
0.1 M	120.0 ± 21.1 ^b^	
**1 M**	**205.0 ± 46.1 ^a^**	
Ionic strength		
**Salt addition (0.2 g)**	**205.0 ± 46.1 ^a^**	solvent: DCM, 1 M acid
No salt	156.0 ± 44.7 ^a^	

(*) DCM—dichloromethane; (**) Values are a mean number of peaks of three replicates ± SD. Different letters (a, b, c) in a column for each parameter represents significantly different (*p* < 0.05) values (Fisher’s Least Significant Difference (LSD), ANOVA) for solvent type and acid molarity and Student *t*-test for ionic strength.

**Table 2:** Validation parameters calculated for a mixture of commercial standards. Compounds are ordered with respect to their increasing retention times.

Retention Time	Compound	Intraday Precision [RSD^*^%]	Interday Precision [RSD%]	Linear Range [μmol/L]	R^2^	LOD ^**^ [μmol/L]	LOQ ^***^ [μmol/L]
9.78	heptanal	9	9	8.857–70.853	0.984	4.4	14.8
13.11	octanal	9	19	8.004–64.035	0.986	4.0	13.3
16.15	nonanal	13	17	7.268–58.142	0.976	3.6	12.1
19.84	decanal	6	16	6.642–53.137	0.983	3.3	11.1
29.70	hexanoic acid	15	15	9.997–79.977	0.985	10.0	33.3
32.51	heptanoic acid	13	26	8.814–70.515	0.988	8.8	29.4
35.05	octanoic acid	11	15	3.944–63.102	0.990	3.9	13.1

(*) Relative standard deviation; (**) LOD - Limit of detection; (***) LOQ - Limit of quantification.

**Table 3:** The number of deconvoluted peaks detected in urine samples from six apparently healthy individuals by GC-MS method.

Urine Sample	Number of Compounds Detected Using GC-MS
1	336
2	326
3	330
4	337
5	338
6	272

## Supplementary Materials

The following are available online, Supplementary Table S1. Tentative assignment of metabolites found to be presented in urine aqueous phase (^1^H-NMR analyses). Supplementary Figure S1. The comparison of the extraction using dichloromethane (DCM), chloroform and diethyl ether. Supplementary Figure S2 ^1^H-NMR 600MHz CPMG spectra of urine obtained from polar phase sample (D2O, T=300K). **1**—2-hydroxyisovaleric acid, **2**—3-methyl-2-oxovaleric acid, **3**—sovaleric acid, **4**—Valine, **5**—3-hydroxyisobutyric acid, **6**—Methylsuccinic acid, **7**—Fucose, **8**—3-hydroxyisovaleric acid, **9**—2-hydroxyisobutyric acid, **10**—2-Phenylpropionic acid, **11**—Alanine, **12**—2-aminoadipic acid, **13**—Acetate, **14**—Acetamide, **15**—Acetone, **16**—Acetoacetic acid, **17**—Succinic acid, **18**—Citrate, **19**—Saccrosine, **20**—Dimethylamine, **21**—Trimethylamine, **22**—*N,N*-dimethylgycine, **23**—*N*-methylhydantoin, **24**—Creatine, **25**—Creatinine, **26**—Choline, **27**—Methanol, **28**—Glycine, **29**—Glycolate, 30—π-methylhistidine, **31**—Trigoneline, **32**—Fumaric acid, **33**—trans-Aconitic acid, **34**—Xanthuretic acid, **35**—Carnosine, **36**—3-Indoxylsulfate, **37**—Imidazole, **38**—Hippurate, **39**—Oxypurinol, **40**—Adenine, **41**—Formic acid, **42**—1-methylnicotinamide.

## References

[1] De Lacy Costello B., Amann A., Al-Kateb H., Flynn C., Filipiak W., Khalid T., Osborne D., Ratcliffe N.M. (2014). A review of the volatiles from the healthy human body. J. Breath Res..

[2] Živković Semren T., Brčić Karačonji I., Safner T., Brajenović N., Tariba Lovaković B., Pizent A. (2018). Gas chromatographic-mass spectrometric analysis of urinary volatile organic metabolites: Optimization of the HS-SPME procedure and sample storage conditions. Talanta.

[3] Bouatra S., Aziat F., Mandal R., Guo A.C., Wilson M.R., Knox C., Bjorndahl T.C., Krishnamurthy R., Saleem F., Liu P. (2013). The Human Urine Metabolome. PLoS ONE.

[4] Monteiro M., Carvalho M., Henrique R., Jerónimo C., Moreira N., De Lourdes Bastos M., De Pinho P.G. (2014). Analysis of volatile human urinary metabolome by solid-phase microextraction in combination with gas chromatography-mass spectrometry for biomarker discovery: Application in a pilot study to discriminate patients with renal cell carcinoma. Eur. J. Cancer.

[5] Jain A., Li X.H., Chen W.N. (2019). An untargeted fecal and urine metabolomics analysis of the interplay between the gut microbiome, diet and human metabolism in Indian and Chinese adults. Sci. Rep..

[6] Zheng L., Wang J., Gao W., Hu C., Wang S., Rong R., Guo Y., Zhu T., Zhu D. (2018). GC/MS-based urine metabolomics analysis of renal allograft recipients with acute rejection. J. Transl. Med..

[7] Zabek A., Paslawski R., Paslawska U., Wojtowicz W., Drozdz K., Polakof S., Podhorska M., Dziegiel P., Mlynarz P., Szuba A. (2017). The influence of different diets on metabolism and atherosclerosis processes—A porcine model: Blood serum, urine and tissues 1H-NMR metabolomics targeted analysis. PLoS ONE.

[8] Taunk K., Taware R., More T.H., Porto-Figueira P., Pereira J.A.M., Mohapatra R., Soneji D., Câmara J.S., Nagarajaram H.A., Rapole S. (2018). A non-invasive approach to explore the discriminatory potential of the urinary volatilome of invasive ductal carcinoma of the breast. RSC Adv..

[9] Taware R., Taunk K., Pereira J.A.M., Dhakne R., Kannan N., Soneji D., Câmara J.S., Nagarajaram H.A., Rapole S. (2017). Investigation of urinary volatomic alterations in head and neck cancer: A non-invasive approach towards diagnosis and prognosis. Metabolomics.

[10] Khalid T., Aggio R., White P., De Lacy Costello B., Persad R., Al-Kateb H., Jones P., Probert C.S., Ratcliffe N. (2015). Urinary volatile organic compounds for the detection of prostate cancer. PLoS ONE.

[11] Antón A.P., Ferreira A.M.C., Pinto C.G., Cordero B.M., Pavón J.L.P. (2014). Headspace generation coupled to gas chromatography-mass spectrometry for the automated determination and quantification of endogenous compounds in urine. Aldehydes as possible markers of oxidative stress. J. Chromatogr. A.

[12] Arasaradnam R.P., Westenbrink E., McFarlane M.J., Harbord R., Chambers S., O’Connell N., Bailey C., Nwokolo C.U., Bardhan K.D., Savage R. (2014). Differentiating coeliac disease from irritable bowel syndrome by urinary volatile organic compound analysis—A pilot study. PLoS ONE.

[13] Arasaradnam R.P., Ouaret N., Thomas M.G., Quraishi N., Heatherington E., Nwokolo C.U., Bardhan K.D., Covington J.A. (2013). A novel tool for noninvasive diagnosis and tracking of patients with inflammatory bowel disease. Inflamm. Bowel Dis..

[14] Drabińska N., Azeem H.A., Krupa-Kozak U. (2018). A targeted metabolomic protocol for quantitative analysis of volatile organic compounds in urine of children with celiac disease. RSC Adv..

[15] Wang M., Xie R., Jia X., Liu R. (2017). Urinary Volatile Organic Compounds as Potential Biomarkers in Idiopathic Membranous Nephropathy. Med. Princ. Pract..

[16] Zhu S., Corsetti S., Wang Q., Li C., Huang Z., Nabi G. (2019). Optical sensory arrays for the detection of urinary bladder cancer-related volatile organic compounds. J. Biophotonics.

[17] Wang Y., Hua L., Jiang J., Xie Y., Hou K., Li Q., Wu C., Li H. (2018). High-pressure photon ionization time-of-flight mass spectrometry combined with dynamic purge-injection for rapid analysis of volatile metabolites in urine. Anal. Chim. Acta.

[18] Zou X., Lu Y., Xia L., Zhang Y., Li A., Wang H., Huang C., Shen C., Chu Y. (2018). Detection of Volatile Organic Compounds in a Drop of Urine by Ultrasonic Nebulization Extraction Proton Transfer Reaction Mass Spectrometry. Anal. Chem..

[19] Batty C.A., Cauchi M., Hunter J.O., Woolner J., Baglin T., Turner C. (2016). Differences in microbial metabolites in urine headspace of subjects with Immune Thrombocytopenia (ITP) detected by volatile organic compound (VOC) analysis and metabolomics. Clin. Chim. Acta.

[20] Drabinska N., Jarocka-Cyrta E., Ratcliffe N.M., Krupa-Kozak U. (2019). The profile of urinary headspace volatile organic compounds after 12-week intake of oligofructose-enriched inulin by children and adolescents with celiac disease on a gluten-free diet: Results of a pilot, randomized, placebo-controlled clinical trial. Molecules.

[21] Aggio R.B.M., Mayor A., Coyle S., Reade S., Khalid T., Ratcliffe N.M., Probert C.S.J. (2016). Freeze-drying: An alternative method for the analysis of volatile organic compounds in the headspace of urine samples using solid phase micro-extraction coupled to gas chromatography—Mass spectrometry. Chem. Cent. J..

[22] Smith S., Burden H., Persad R., Whittington K., De Lacy Costello B., Ratcliffe N.M., Probert C.S. (2008). A comparative study of the analysis of human urine headspace using gas chromatography-mass spectrometry. J. Breath Res..

[23] O’Lenick C.R., Pleil J.D., Stiegel M.A., Sobus J.R., Wallace M.A.G. (2018). Detection and analysis of endogenous polar volatile organic compounds (PVOCs) in urine for human exposome research. Biomarkers.

[24] Alves A.A.R., Barros E.B.P., Rezende C.M., Ferreira V., Lopez R.B.T.-F.S. (2014). Chapter 78—Method Development and Optimization of Liquid–Liquid Extraction for the Quantitative Analysis of Volatile Compounds from Brazilian Grape Juices. Flavour Science Proceedings from XIII Weurman Flavour Research Symposium.

[25] Orak H.H., Bahrisefit I.S., Sabudak T. (2019). Antioxidant Activity of Extracts of Soursop (Annona muricata L.) Leaves, Fruit Pulps, Peels, and Seeds. Polish J. Food Nutr. Sci..

[26] Soliman M.A., Pedersen J.A., Suffet I.M. (2004). Rapid gas chromatography–mass spectrometry screening method for human pharmaceuticals, hormones, antioxidants and plasticizers in water. J. Chromatogr. A.

[27] Kataoka H. (2003). New trends in sample preparation for clinical and pharmaceutical analysis. TrAC Trends Anal. Chem..

[28] Zlatkis A., Liebich H.M. (1971). Profile of Volatile Metabolites in Human Urine. Clin. Chem..

[29] Seyler T.H., Kim J.G., Hodgson J.A., Cowan E.A., Blount B.C., Wang L. (2013). Quantitation of Urinary Volatile Nitrosamines from Exposure to Tobacco Smoke*. J. Anal. Toxicol..

[30] Cozzolino R., De Magistris L., Saggese P., Stocchero M., Martignetti A., Di Stasio M., Malorni A., Marotta R., Boscaino F., Malorni L. (2014). Use of solid-phase microextraction coupled to gas chromatography-mass spectrometry for determination of urinary volatile organic compounds in autistic children compared with healthy controls. Anal. Bioanal. Chem..

[31] Cequier-Sánchez E., Rodríguez C., Ravelo Á.G., Zárate R. (2008). Dichloromethane as a Solvent for Lipid Extraction and Assessment of Lipid Classes and Fatty Acids from Samples of Different Natures. J. Agric. Food Chem..

[32] Ciska E., Drabińska N., Honke J., Narwojsz A. (2015). Boiled Brussels sprouts: A rich source of glucosinolates and the corresponding nitriles. J. Funct. Foods.

[33] Risticevic S., Lord H., Górecki T., Arthur C.L., Pawliszyn J. (2010). Protocol for solid-phase microextraction method development. Nat. Protoc..

[34] Naz S., Vallejo M., García A., Barbas C. (2014). Method validation strategies involved in non-targeted metabolomics. J. Chromatogr. A.

[35] Kordalewska M., Macioszek S., Wawrzyniak R., Sikorska-Wiśniewska M., Śledziński T., Chmielewski M., Mika A., Markuszewski M.J. (2019). Multiplatform metabolomics provides insight into the molecular basis of chronic kidney disease. J. Chromatogr. B.

[36] Rosen Vollmar A.K., Rattray N.J.W., Cai Y., Santos-Neto Á.J., Deziel N.C., Jukic A.M.Z., Johnson C.H. (2019). Normalizing Untargeted Periconceptional Urinary Metabolomics Data: A Comparison of Approaches. Metabolites.

[37] Rocha S.M., Caldeira M., Carrola J., Santos M., Cruz N., Duarte I.F. (2012). Exploring the human urine metabolomic potentialities by comprehensive two-dimensional gas chromatography coupled to time of flight mass spectrometry. J. Chromatogr. A.

[38] Görling B., Bräse S., Luy B. (2016). NMR chemical shift ranges of urine metabolites in various organic solvents. Metabolites.

[39] Fathi F., Brun A., Rott K.H., Cobra P.F., Tonelli M., Eghbalnia H.R., Caviedes-Vidal E., Karasov W.H., Markley J.L. (2017). NMR-based identification of metabolites in polar and non-polar extracts of avian liver. Metabolites.

[40] Silva C.L., Passos M., Cmara J.S. (2011). Investigation of urinary volatile organic metabolites as potential cancer biomarkers by solid-phase microextraction in combination with gas chromatography-mass spectrometry. Br. J. Cancer.

