# Supplementary material for: An Optimization of Liquid–Liquid Extraction of Urinary Volatile and Semi-Volatile Compounds and Its Application for Gas Chromatography-Mass Spectrometry and Proton Nuclear Magnetic Resonance Spectroscopy
Source: Molecules. 2020 Aug 11;25(16):3651. doi: 10.3390/molecules25163651 (PMC7463579; doi:10.3390/molecules25163651)
Supplement: Supplementary file 1 [file molecules-25-03651-s001.pdf]

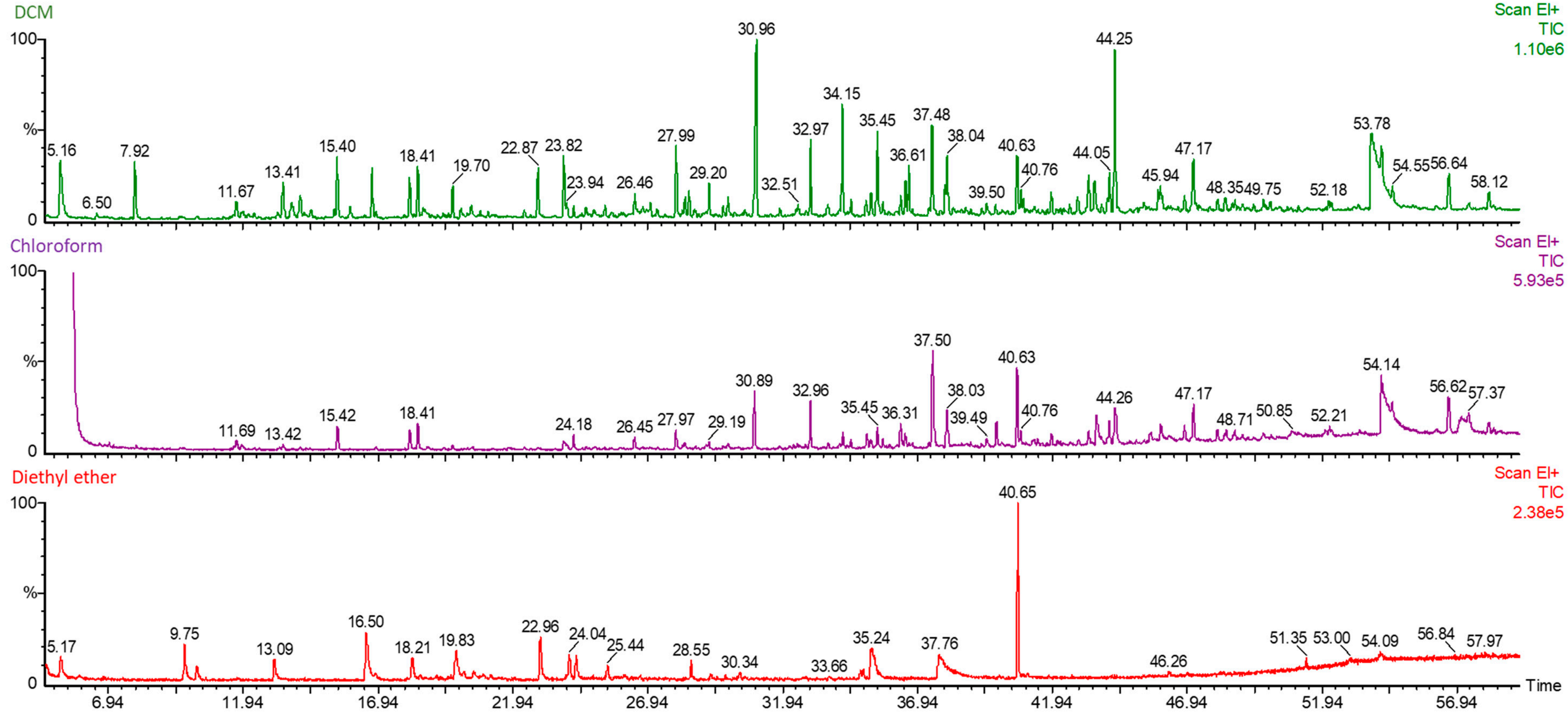

Supplementary Figure A1. The comparison of the extraction using dichloromethane (DCM), chloroform and diethyl ether.

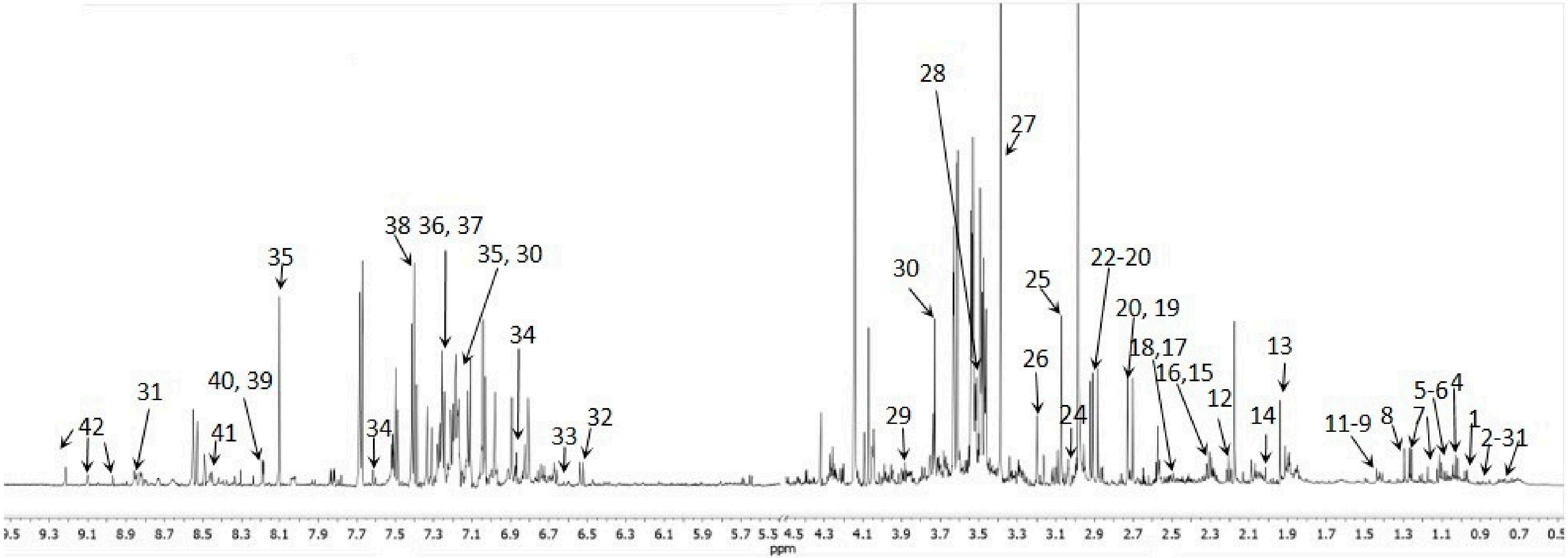

Supplementary Figure A2. ^{1}H NMR 600MHz CPMG spectra of urine obtained from polar phase (D_2O, T=300K)
1 – 2-hydroxyisovaleric acid, 2 – 3-methyl-2-oxovaleric acid, 3 – sovaleric acid, 4 – Valine, 5 – 3-hydroxyisobutyric acid, 6 – Methylsuccinic acid, 7 – Fucose, 8 – 3-hydroxyisovaleric acid, 9 – 2-hydroxyisobutyric acid, 10 – 2-Phenylpropionic acid, 11 – Alanine, 12 – 2-aminoadipic acid, 13 – Acetate, 14 – Acetamide, 15 – Acetone, 16 – Acetoacetic acid, 17 – Succinic acid, 18 – Citrate, 19 – Saccrosine, 20 – Dimethylamine, 21 – Trimethylamine, 22 – N,N-dimethylgycine, 23 – N- methylhydantoin 24 – Creatine, 25 – Creatinine, 26 – Choline, 27 – Methanol, 28 – Glycine, 29 – Glycolate, 30 – π-methylhistidine, 31 – Trigoneline, 32 – Fumaric acid, 33 – trans-Aconitic acid, 34 – Xanthuretic acid, 35 – Carnosine, 36 – 3-Indoxylsulfate, 37 – Imidazole, 38 – Hippurate, 39 – Oxypurinol, 40 – Adenine, 41 – Formic acid, 42 – 1-methylnicotinamide.

Supplementary Table A1. Tentative assignment of metabolites found to be presented in urine aqueous phase (NMR analyses).

Nr	Name	Chemical Shift [ppm]
1.	2-hydroxyisovaleric acid	0.8 (d); 0.95(d); 3.84 (d)
2.	3-methyl-2-oxovaleric acid	0.87(t); 1.09(d); 1.68 (m)
3.	Isovaleric acid	0.89(d)
4.	Valine	1.01(t)
5.	3-hydroxyisobutyric acid	1.05(d); 2.47(m)
6.	Methylsuccinic acid	1.07(d); 2.5(dd)
7.	Fucose	1.2(d); 1.24(d); 3.4(m); 3.6(t); 3.78(m); 3.83(dd); 4.5(d); 5.2(d)
8.	3-hydroxyisovaleric acid	1.27(s); 2.34(s)
9.	2-hydroxyisobutyric acid	1.35(s)
10.	2-Phenylpropionic acid	1.4(d); 3.6(q); 7.3(m); 7.4(m)
11.	Alanine	1.48(d); 3.5(q)
12.	2-aminoadipic acid	1.58(m); 1.63(m); 1.81(m); 1.88(m); 2.18(t); 3.73(m)
13.	Acetate	1.92(s)
14.	Acetamide	2.0(s); 5.8; 7.5
15.	Acetone	2.25(s)
16.	Acetoacetic acid	2.27(s)
17.	Succinic acid	2.39(s)
18.	Citrate	2.5(d); 2.68(d)
19.	Saccrosine	2.69(s); 3.6(s)
20.	Dimethylamine	2.71(s)
21.	Trimethylamine	2.87(s)
22.	N,N-dimethylgycine	2.89(s); 3.7(s)
23.	N-methylhydantoin	2.91(s); 4.08(s)

24.	Creatine	3.02(s); 3.94(s)
25.	Creatinine	3.06(s); 4.055(s)
26.	Choline	3.18(s)
27.	Methanol	3.37(s)
28.	Glycine	3.51(s)
29.	Glycolate	3.91(s)
30.	π-methylhistidine	3.2(dd); 3.3(dd); 3.72(s); 3.96(q); 7.09(s)
31.	Trigoneline	4.42(s); 8.06(t); 8.78(d); 8.80(d); 9.08(s)
32.	Fumaric acid	6.50(s)
33.	trans-Aconitic acid	6.58(s)
34.	Xanthuretic acid	6.87(s); 7.18(d); 7.59(d)
35.	Carnosine	4.5(m); 7.11(s); 8.1(s)
36.	3-Indoxylsulfate	7.17(t); 7.24(t); 7.32(s); 7.39(d); 7.66(d)
37.	Imidazole	7.29(b.s); 8.31(b.s)
38.	Hippurate	3,96(d); 7.44(t); 7.62(m); 7.82(d); 8.5(b.s)
39.	Oxypurinol	8.17(s)
40.	Adenine	8.19(s); 8.23(s)
41.	Formic acid	8.43(s)
42.	1-methylnicotinamide	9.2 (s); 8.95 (s); 8.83 (s); 8.17(q); 4.47(s)
